# Cross-neutralization of distant coronaviruses correlates with Spike S2-specific antibodies from immunocompetent and immunocompromised vaccinated SARS-CoV-2-infected patients

**DOI:** 10.21203/rs.3.rs-5487774/v1

**Published:** 2024-12-05

**Authors:** Sara V. Patel, Brooke M. Leeman, Patricia J. Botros, Joanna Folta, Dhiman Shahid, Anya I. Rocque, Andrew S. Joyal, Joseph A. Vecchio, Eliza Passell, Dessie Tien, Zahra Reynolds, Karry Su, Tammy D. Vyas, Jatin M. Vyas, Emory Abar, Mamadou Barry, Andrew Alexandrescu, Zachary Wallace, Jeffrey M. DaCosta, Manish C. Choudhary, Trevor Tamura, Gregory Edelstein, Yijia Li, Rinki Deo, Jeffrey A. Sparks, Julie Boucau, Owen Glover, Amy Barczak, Jacob Lemieux, Mark J. Siedner, Jonathan Z. Li, Ismael Ben Fofana

**Affiliations:** 1: Biology Department, Boston College, 140 Commonwealth Avenue, Chestnut Hill, MA; 2: Massachusetts General Hospital, Harvard Medical School, Cambridge, MA, USA; 3: Brigham and Women’s Hospital, Harvard Medical School, Boston, MA, USA.; 4: Ragon Institute of MGH, MIT, and Harvard, Boston, MA, USA.

## Abstract

As of May 2023, the public health emergency of COVID-19 was lifted across the globe. However, SARS-CoV-2 infections continue to be recorded worldwide. This situation has been attributed to the ability of the virus to evade host immune responses including neutralizing antibody-derived Immunity. The vast majority of antibody escape mutations have been associated with the S1 subunit of the spike protein, especially the Receptor Binding Domain (RBD) but also the N-terminal Domain (NTD). The other region of the spike, the S2 subunit, is the most conserved region amongst coronaviruses. We hypothesized that S2-specific antibody responses are suboptimal in vaccinated and SARS-CoV-2 infected patients resulting in an ineffective neutralization of distant coronaviruses. Here, we analyzed S2-specific antibody responses SARS-CoV-2-infected individuals, including a mixed cohort of those with and without immunosuppression and prior vaccination. We found that S2-specific antibody responses are generally lower than S1-specific antibody responses. Furthermore, we observed in immunocompetent individuals that S1 and S2-specific antibody responses are both positively correlated with Wuhan, Omicron, SARS-CoV and W1V1-CoV pseudovirus neutralization. Among the immunocompromised patients, S1-specific antibody responses were rarely correlated with pseudovirus neutralization in contrast to S2-specific antibody responses which frequently correlated with pseudovirus neutralization. These data highlight the potential of the S2-subunit as an ideal target for induction of cross-neutralizing antibody immunity against divergent coronaviruses.

## Introduction

Coronavirus Disease of 2019 (COVID-19) emerged in late 2019 in Wuhan (China) [1] and rapidly expanded across the globe with the World Health Organization (WHO) declaring a global health pandemic in March 2020 [2]. A number of vaccine candidates were developed including Pfizer-BioNTech, Moderna with emergency use authorization granted in the United States under a 1-year period [3]. Millions of people were immunized worldwide and many lives were saved [4]. In the US the public health emergency of COVID-19 expired in May, 2023 [5]. However, the virus has continued to evolve and circulate across the globe with occurrences of variants of concern including the two major waves of the Delta and Omicron variants ongoing [6]. This raises a concern for vaccine-induced protection amongst the most vulnerable sections of the population, especially the immunocompromised [7–10].

The titer of neutralizing antibodies against SARS-CoV-2 has been identified as a predictor of COVID-19 disease severity [11]. COVID-19 disease severity has also been associated with high levels of SARS-CoV-2 antibodies in other studies which did not always discriminate between binding antibody titers and neutralization potency [12, 13]. SARS-CoV-2 ability to evade antibody responses through S1 region mutations including the receptor binding domain has been described [11, 14–17]. It is noteworthy that SARS-CoV-2 ability to evade antibody neutralization is not limited to the RBD [18]. Nucleotide deletions were observed in the N-terminal Domain (NTD) of the S1-subunit of the spike protein leading to escape from neutralizing antibody responses [18].

The ability of SARS-CoV-2 to escape antibody responses and the well-established importance of neutralization antibody responses to disease progression and survival has enhanced the concerns for immunocompromised individuals [7–10]. Here, we measured the levels of antibody responses targeting the S1 and S2 regions of the spike from serum of vaccinated individuals with non-severe COVID-19, including those with and without immunosuppression. We then analyzed correlations between neutralization of wild-type Wuhan, Omicron BA.1, SARS-CoV and W1V1-CoV pseudoviruses. We found that S1-specific antibody responses are more abundant while S2-specific antibody responses are suboptimal for efficient neutralization of distant coronaviruses, SARS-CoV and W1V1-CoV. Nonetheless, we found that S1- and S2-specific antibody levels were generally correlated with pseudovirus neutralization for both immunocompetent and immunocompromised patients. A more restrictive pattern emerged with the immunocompromised for whom S2-specific antibody levels distinctively correlated with Omicron, SARS-CoV and W1V1-CoV pseudovirus neutralization.

## Results

Study specimens were tested from individuals enrolled in the POSITIVES study (Post-vaccination Viral Characteristics Study), a prospective observational cohort study that enrolls ambulatory persons with acute COVID-19. Specimens were collected between January 2021 and May 2023 [19]. SARS-CoV-2 infection was confirmed by RT-PCR. Eight of the 87 participants were non-vaccinated and one participant received a single dose of the Johnson & Johnson COVID-19 vaccine. All other participants received between 2 and 5 doses of either the Moderna or Pfizer/BioNTech COVID-19 mRNA vaccines. All 3 vaccines at the time of this analysis were based on a Wuhan spike trimer formulation. Table 1 presents the demographic and clinical characteristics of the participants.

### Validity of modified soluble S2 as ELISA antigens

We first sought to measure antibody levels against soluble S1 and S2 antigens. The amino-acid sequences corresponded to the wild-type Wuhan and the Omicron B.1.1.529 variant (also known as the Omicron BA.1 variant and hereafter referred to as Omicron were compared to the soluble Wuhan S2 and Omicron S2 mutated for solubility by the manufacturer (Acro Biosystem). The analysis also included the S1 and S2 subunits corresponding to SARS coronavirus (SARS-CoV) and the bat SARS-Like coronavirus W1V1 (W1V1-CoV).

The soluble Wuhan S2 and Omicron S2 antigens were similar and clustered to their corresponding wild-type Wuhan S2 and Omicron S2 subunits (Supplementary Figure 1). In general, comparison of S1 and S2 subunits confirmed the similarities between Wuhan and Omicron and their isolation from the distant relatives SARS-CoV and W1V1-CoV (Supplementary Tables 1–3).

### S1-specific antibody titers surpassed S2-specific antibody levels

**Figure 1:**
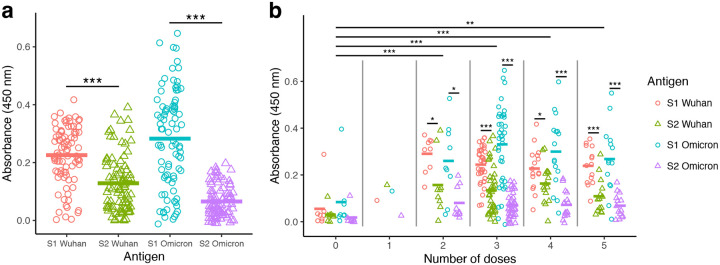
High S1-specific and low S2-specific antibody levels were observed with vaccinated and SARS-CoV-2-infected individuals. Binding antibody levels were measured using serum obtained from 87 study participants. Serum binding antibody titers were measured by ELISA using S1-Wuhan, S2-Wuhan, S1-Omicron, and S2-Omicron as antigens. Antibodies were detected using secondary anti-human IgG-HRP conjugated. Absorbance was determined at 450 nm. (a) S1- and S2-specific antibodies levels from all participants. Paired *t*-tests were used to compare antibody absorbance for S1 vs S2 regions of Wuhan and Omicron antigens. (b) S1- and S2-specific antibodies levels from all participants according to the number of vaccine doses. For each dose number, paired *t*-tests were used to compare antibody absorbance for S1 vs S2 regions of Wuhan and Omicron antigens. Means for each number of doses were compared using a linear mixed model with individual patient identification as a random effect. Statistical significance was defined as *P < 0.05, **P < 0.01, and *** *P* < 0.001.

Antibody levels were measured by ELISA using the S1 and S2 antigens from the Wuhan and Omicron strains in the form of soluble proteins (Figure 1). S1-Wuhan-specific antibody levels (mean=0.23) were significantly higher than S2-Wuhan-specific antibody levels (mean=0.13) (paired t-test; *P* < 0.001) (Figure 1a). The same pattern was seen for Omicron, with the S1-Omicron-specific antibody levels (mean=0.28) significantly higher than S2-Omicron-specific antibody levels (mean=0.07) (paired *t*-test; *P* < 0.001) (Figure 1a). Overall, S1-Omicron-specific antibody levels were the highest while S2-Omicron-specific antibody levels were the lowest (Figure 1a).

### Binding antibody titers were higher with vaccinated groups compared to the non-vaccinated group, with no significant differences among the booster groups (2–5 doses)

Samples were subsequently analyzed according to the vaccination status and number of doses for comparison of S1- and S2-specific antibody levels. Vaccinated participants (1–5 doses; n=79) presented higher antibody titers than non-vaccinated ones (0 doses; n=8) (Figure 1b). The 1-dose vaccination group had only 1 participant, which reduced the power of statistical analyses involving this group. The remaining vaccination groups (doses 2–5) had significantly higher antigen binding compared to non-vaccinated participants (linear mixed model with Tukey HSD post-hoc test; *P* < 0.01). However, no significant differences in antibody levels were observed in comparisons between the multiple booster groups (2 to 5 doses; Figure 1b).

Inter-group analyses were followed up with intra-group analyses, in which paired t-tests were used to test if participants had higher antibody levels for S1- or S2-specific antibodies. For groups with either 0 or 1 dose, no significant differences were found since low sample size reduced statistical power (Figure 1b). For groups with 2–5 doses, all tests found significantly higher antibody levels in S1 compared to S2-specific antigens. For example, in participants that received 3 doses (n=39), S1-Wuhan antibody levels (mean=0.24) were significantly higher than S2-Wuhan levels (mean=0.13) (paired *t*-test; *P* < 0.001), and also S1-Omicron antibody levels (mean=0.33) were significantly higher than S2-Omicron levels (mean=0.07) (paired *t*-test; *P* < 0.001) (Figure 1b).

### Pseudovirus neutralization was higher with Wuhan and Omicron compared to SARS-CoV and W1V1-CoV

Sera from all participants (n=87) were evaluated for neutralization capacity of several coronaviruses. We used a previously published pseudovirus-based neutralization assay [11, 14, 20] with pseudoviruses corresponding to the Wuhan and Omicron variants, as well as the distant relatives SARS-CoV and W1V1-CoV [21].

**Figure 2:**
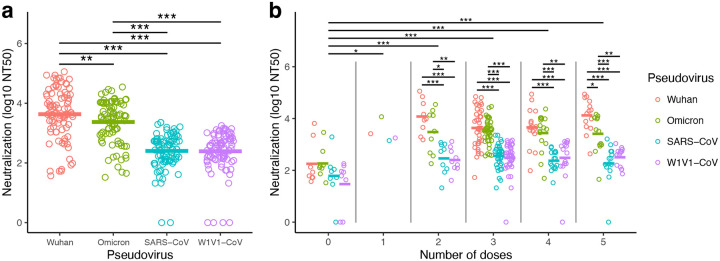
Highest antibody neutralization concentrations were observed against Wuhan and Omicron pseudoviruses. Neutralizing antibody levels were measured using serum obtained from 87 study participants. Neutralization concentrations were reported as a 50% neutralization titer (NT50). Values were transformed due to skew in the data (log10 of NT50+1 transformation). (a) Neutralization titers for Wuhan, Omicron, SARS-CoV, and W1V1-CoV pseudoviruses. Titer means of pseudoviruses were compared using a linear mixed model with individual patient identification as a random effect. (b) Neutralization titers analyzed according to the number of vaccine doses. Similar linear mixed models were used to compare values within and among the number of doses. Statistical significance was defined as *P < 0.05, **P < 0.01, and *** *P* < 0.001.

Highest serum antibody neutralization concentrations were observed with Wuhan pseudovirus (Figure 2). Nine of the samples required a higher serum starting dilution of 20x and 3 samples necessitated a higher serum starting dilution of 30x for the crossing of the 50% neutralization levels in order to generate the NT50 values (Supplementary Figure 2). Overall, strong serum antibody neutralization concentrations were observed against the Omicron pseudovirus, but these were significantly lower when compared to the Wuhan pseudovirus neutralization (log_10_ NT50+1 transformation; Wuhan mean=3.63, Omicron B1.1.259 mean=3.37; linear mixed model with Tukey HSD post-hoc test; *P* < 0.01, Figure 2a).

Lower serum neutralization concentrations were observed with the more distantly related SARS-CoV (log_10_ NT50+1 transformation; mean=2.40) and W1V1-CoV (mean=2.39) pseudoviruses. Wuhan pseudovirus neutralization concentrations were significantly higher than those observed with both SARS-CoV (linear mixed model with Tukey HSD post-hoc test; *P* < 0.001) and W1V1-CoV (linear mixed model; *P* < 0.001) (Figure 2a). Similar results were recovered when comparing Omicron pseudovirus neutralization concentrations to those with SARS-CoV and W1V1-CoV (linear mixed model with Tukey HSD post-hoc test; *P* < 0.001; Figure 2a). No significant differences were observed between SARS-CoV and W1V1-CoV pseudovirus neutralization (*P* > 0.05; Figure 2a).

### Vaccination significantly improved pseudovirus neutralization potency

Vaccinated participants (1–5 doses; n=79) presented higher neutralization concentrations compared to non-vaccinated participants (0 doses; n=8) (Figure 2b). Neutralization was lowest in the 0-dose group (log10 NT50+1 transformation; mean=1.94), and the means of all other dose groups were significantly higher. This was found even for the comparison to the 1-dose group that included only one participant (mean=3.47; linear mixed model with Tukey HSD test; *P* < 0.05). Neutralization was further elevated in participants that had boosters (2-dose mean=3.10; 3-dose mean=3.05; 4-dose mean=2.99; 5-dose mean=3.08). Comparisons of the non-vaccinated (0-dose) to these groups with boosters (2–5 dose groups) were all highly significant (linear mixed model with Tukey HSD test; *P* < 0.001; Figure 2b). Intra-group analyses (i.e., within those with the same number of vaccine doses) did not find differences among pseudovirus neutralization for those within the 0-dose and 1-dose groups. Within groups that had multiple doses (2–5 doses), pseudovirus neutralization was significantly higher for Wuhan and Omicron compared to SARS-CoV and W1V1-CoV (linear mixed model with Tukey HSD test; all *P* < 0.05; Figure 2b).

### No significant correlation was observed between antibody responses and the number of days from the last dose

We hypothesized that the time since last immunization could impact antibody responses. We first investigated if the number of days after the last vaccine dose influenced the level of antibody responses in the vaccinated participants (Supplementary Figure 3). The single dose participant was at 262 days after his immunization while, as expected, the group with 2 doses had the most extended period (mean=393) followed by 3 doses (mean=201.1), 4 doses (mean=148.8) and 5 doses (mean=134.9) (Supplementary Figure 3a). Significant differences were only observed in comparisons with the 2-dose and the 3-, 4-, and 5-dose groups (one-way ANOVA with Tukey HSD test; *P* < 0.001; Supplementary Figure 3a). However, no significant correlations were observed between the number of days from the last vaccine and S1- or S2-specific antibody levels (Pearson’s correlation; *P* > 0.05; Supplementary Figure 3b). The potential impact of the number of days after the last vaccine dose on the pseudovirus neutralization concentrations produced similarly low correlation coefficients, with the one significant result of a significant negative correlation between number of days since last dose and neutralization of SARS-CoV (Pearson’s correlation; P < 0.05; Supplementary Figure 3c).

### S1- and S2-specific antibody titers generally correlated positively with pseudovirus neutralization potency

We next evaluated the relationship between antigen-specific antibody titers and neutralization capacity. Linear correlations between pseudovirus neutralization concentrations and S1- or S2- specific Wuhan/Omicron antibody levels were analyzed for all participants (n=87). Separate analyses were done for each pseudovirus (Wuhan, Omicron, SARS-CoV and W1V1-CoV), resulting in 16 comparisons.

**Figure 3:**
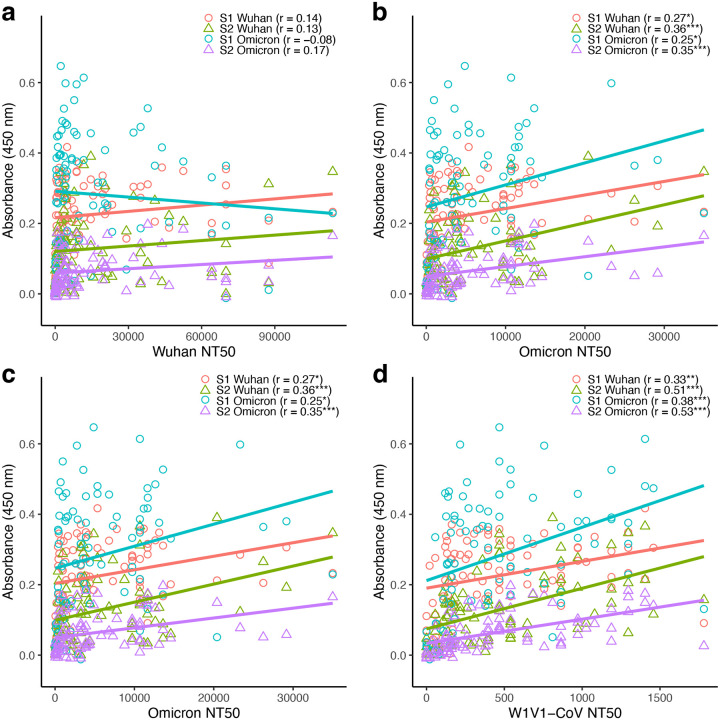
Antibody titers were predominantly positively correlated to pseudovirus neutralization. Binding (measured by ELISA) and neutralizing (measured as 50% neutralization titer; NT50) antibody levels were compared for the 87 study participants. (a) S1-Wuhan, S2-Wuhan, S1-Omicron and S2-Omicron specific levels vs Wuhan pseudovirus NT50. (b) S1-Wuhan, S2-Wuhan, S1-Omicron, and S2-Omicron specific levels vs Omicron pseudovirus NT50. (c) S1-Wuhan, S2-Wuhan, S1-Omicron, and S2-Omicron specific levels vs SARS-CoV pseudovirus NT50. (d) S1-Wuhan, S2-Wuhan, S1-Omicron, and S2-Omicron specific levels vs W1V1-CoV pseudovirus NT50. Correlations were analyzed using Pearson’s correlation, with statistical significance defined as *P < 0.05, **P < 0.01, and ***P < 0.001.

There was a predominantly positive correlation between neutralization capacity and antibody titers, with 15 of the 16 analyses resulting in a positive correlation coefficient (Figure 3). Furthermore, 12 or the 16 analyses had significantly positive coefficients (Pearson’s correlation; *P* < 0.05). Antibody correlations with the Wuhan pseudovirus were the weakest, with correlations between −0.08 and 0.17 and no significant results (Figure 3a). For the remaining pseudoviruses, significant positive correlations were recovered in all analyses (Figure 3b–d). The highest correlation coefficients were found in analyses with W1V1-CoV, with *r*=0.53 for S2-Omicron and *r*=0.51 for S2-Wuhan (both *P* < 0.001; Figure 3d).

Although S1-specific antibody levels were higher compared to their S2-specific counterparts (i.e., higher y-intercepts), the correlation between neutralization and antibody production was mostly higher in S2- compared to S1-specific analyses. For example, for the Omicron pseudovirus the correlation coefficient was higher for S2-Wuhan (*r* = 0.36) compared to S1-Wuhan (*r* = 0.27), as well as for S2-Omicron (*r* = 0.35) compared to S1-Omicron (*r* = 0.25) (Figure 5b). This pattern was found for 6 of the 8 possible S1- vs S2-specific comparisons.

### Antibody responses observed with the immunocompetent participants were generally higher than that of the immunocompromised participants

**Figure 4:**
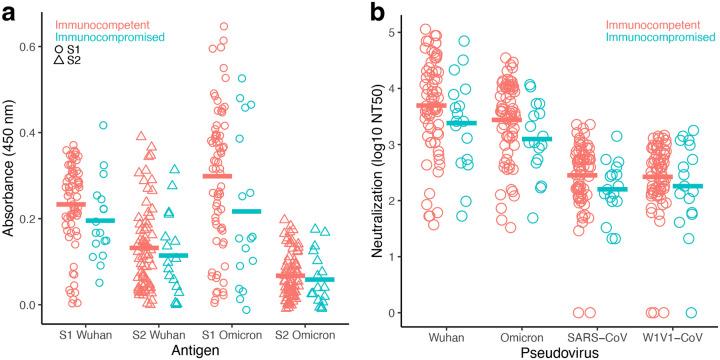
Lower, but not significant, binding and neutralization antibody levels were observed with the immunocompromised individuals. Binding and neutralizing antibody levels compared for 70 immunocompetent and 17 immunocompromised study participants. NT50 values were transformed due to skew in the data (log10 of NT50+1 transformation). (a) S1- and S2-specific antibody levels measured by ELISA using S1-Wuhan, S2-Wuhan, S1-Omicron, and S2-Omicron as antigens. (b) Serum antibody neutralizing concentrations (50% neutralization titer; NT50) were determined against Wuhan, Omicron, SARS-CoV and W1V1-CoV pseudoviruses. For each measurement, immunocompetent and immunocompromised means were analyzed using a *t*-test. Statistical significance was defined as *P < 0.05, **P < 0.01, and *** *P* < 0.001.

Since the POSITIVES cohort includes immunocompetent as well as immunocompromised participants, we evaluated the impact of immunocompromised status on coronavirus-specific humoral immunity. In this sub-study, 17 of the 87 samples were obtained from immunocompromised participants. Binding (Figure 4a) and neutralizing (Figure 4b) antibody titers were overall lower for the immunocompromised, although no comparison reached statistical significance.

### Antibody levels and pseudovirus neutralization were correlated more strongly in immunocompetent patients than in the immunocompromised group

**Figure 5:**
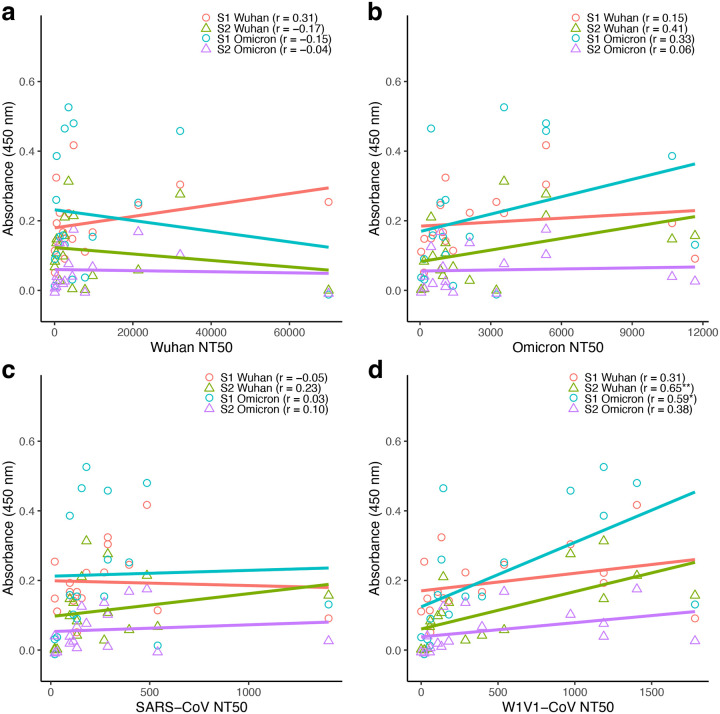
Antibody titers were generally positively, but not significantly, correlated to pseudovirus neutralization in immunocompromised participants. Binding (measured by ELISA) and neutralizing (measured as 50% neutralization titer; NT50) antibody levels were compared for the 17 immunocompromised participants. (a) S1-Wuhan, S2-Wuhan, S1-Omicron, and S2-Omicron specific levels vs Wuhan pseudovirus NT50. (b) S1-Wuhan, S2-Wuhan, S1-Omicron, and S2-Omicron specific levels vs Omicron pseudovirus NT50. (c) S1-Wuhan, S2-Wuhan, S1-Omicron, and S2-Omicron specific levels vs SARS-CoV pseudovirus NT50. (d) S1-Wuhan, S2-Wuhan, S1-Omicron, and S2-Omicron specific levels vs W1V1-CoV pseudovirus NT50. Correlations were analyzed using Pearson’s correlation, with statistical significance defined as *P < 0.05, **P < 0.01, and ***P < 0.001.

Correlations between antibody levels and pseudovirus neutralization titers were analyzed separately for immunocompromised and immunocompetent groups. In each case, S1- and S2-specific Wuhan and Omicron antibodies were tested with each pseudovirus in a separate analysis. For the immunocompromised group (Figure 5), 12 of the 16 correlations were positive, but only two tests were significant. For the W1V1-CoV pseudovirus, there was a significant positive correlation between neutralization and S2-Wuhan (Pearson’s correlation; *r* = 0.65; *P* < 0.01) and S1 Omicron (Pearson’s correlation; *r* = 0.59; *P* < 0.05) antibody levels.

**Figure 6:**
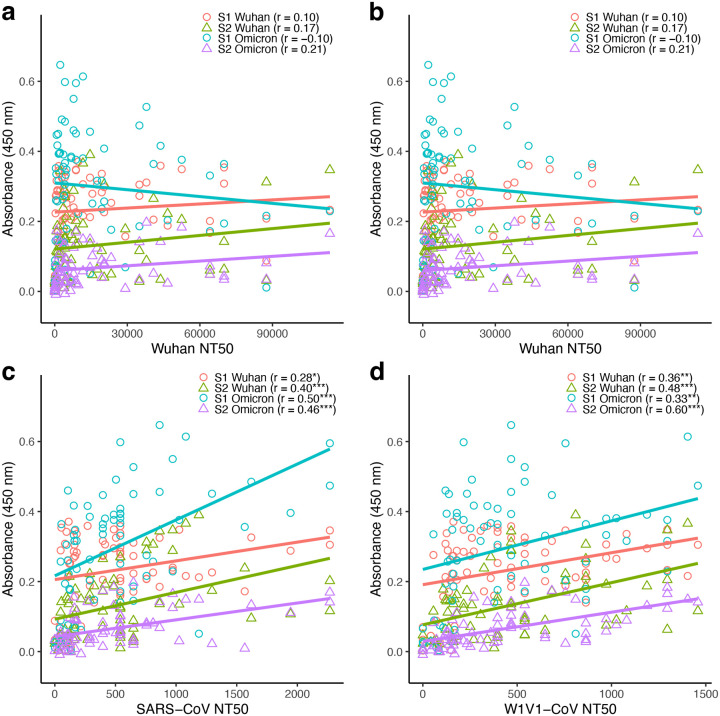
Antibody titers were predominantly positively and significantly correlated to pseudovirus neutralization in immunocompetent participants. Binding (measured by ELISA) and neutralizing (measured as 50% neutralization titer; NT50) antibody levels were compared for the 70 immunocompetent participants. (a) S1-Wuhan, S2-Wuhan, S1-Omicron, and S2-Omicron specific levels vs Wuhan pseudovirus NT50. (b) S1-Wuhan, S2-Wuhan, S1-Omicron, and S2-Omicron specific levels vs Omicron pseudovirus NT50. (c) S1-Wuhan, S2-Wuhan, S1-Omicron, and S2-Omicron specific levels vs SARS-CoV pseudovirus NT50. (d) S1-Wuhan, S2-Wuhan, S1-Omicron, and S2-Omicron specific levels vs W1V1-CoV pseudovirus NT50. Correlations were analyzed using nonparametric Spearman correlation on GrapPad prism. Correlations were analyzed using Pearson’s correlation, with statistical significance defined as *P < 0.05, **P < 0.01, and ***P < 0.001.

Correlations between neutralization and antibody levels were considerably stronger in the immunocompetent group (Figure 6) which had a larger sample size (n=70) than the immunocompromised (n=17). Here, 15 of the 16 correlations were positive, with 10 being significantly so. For the Wuhan pseudovirus there were no significant correlations with S1- and S2-specific antibody levels (Pearson’s correlation; *P* > 0.05; Figure 6a). For the Omicron pseudovirus there were significant positive correlations for S2-Wuhan (Pearson’s correlation; *r* = 0.36; *P* < 0.01) and S2-Omicron (*r* = 0.39; *P* < 0.001) antibody levels (Figure 6b). For both SARS-CoV and W1V1-CoV pseudoviruses, significant positive correlations were found in all tests (Figure 6c–d). As in the analyses with all samples, the same general pattern of higher correlations between neutralization and antibody production for S2- compared to S1-specific counterpart was found when only analyzing immunocompetent participants.

### Increasing the number of booster doses significantly improved antibody responses for immunocompetent, but not immunocompromised, participants

The impact of booster doses on the levels of antibody responses was examined separately for the immunocompromised and immunocompetent participants (Supplementary Figure 4). All the immunocompromised participants received at least one vaccine dose so group comparisons were limited to only vaccinated participants (Supplementary Figure 4a). Although higher S1- and S2-specific antibody levels were observed for 3-, 4-, and 5-dose groups, the means of different dose groups were not significantly different from each other (linear mixed model with Tukey HSD test; *P* > 0.05; Supplementary Figure 4a). In analyses within each dose group of immunocompromised participants, significantly higher antibody levels were found in the 4-dose S1- vs S2 Omicron (paired t-test; *P* < 0.05), the 5-dose S1- vs S2-Wuhan (paired t-test; *P* < 0.05), and the 5-dose S1- vs S2-Omicron (paired t-test; *P* < 0.001), comparisons (Supplementary Figure 4a).

In the immunocompetent group, multiple doses of vaccines promoted higher antibody production than the non-vaccinated while no significant difference was observed between the multiple doses (doses 2–5; Supplementary Figure 4b). There were no immunocompetent participants that received only one dose, but those receiving 2, 3, 4, and 5 doses all had higher mean antibody levels compared to non-vaccinated immunocompetent participants (linear mixed model with Tukey HSD test; *P* < 0.001). There were no statistical differences among mean antibody levels in the 2-, 3-, 4-, and 5-dose groups (*P* > 0.05). Within each immunocompetent dose group, paired *t*-tests were used to evaluate if S1- and S2-specific antigens had equal means. For the 0-dose group there were no significant differences, but for most comparisons in the other dose groups there were higher antibody levels toward the S1-specific antigen compared to its S2-specific counterpart (6 of 8 comparisons; *P* < 0.01).

### Increasing the number of booster doses did not significantly improved neutralization titers observed with the immunocompromised participants

We also separately examined the impact of booster doses on pseudovirus neutralization for the immunocompromised and immunocompetent participants and recovered similar results (Supplementary Figure 5). Mean neutralization levels among the dose groups did not differ significantly (linear mixed model with Tukey HSD test; *P* > 0.05; Supplementary Figure 5a). Neutralization was also similar across the different pseudoviruses for within dose group comparisons with three exceptions: Wuhan vs SARS-CoV in the 3-dose group, Wuhan vs SARS-CoV in the 5-dose group, and Wuhan vs W1V1-CoV in the 5-dose group (Supplementary Figure 6a).

In contrast again, an increased number of vaccine doses promoted a stronger response in the immunocompetent group (Supplementary Figure 5b). Those receiving 2, 3, 4, and 5 doses all had higher neutralization levels compared to non-vaccinated immunocompetent participants (linear mixed model with Tukey HSD test; *P* < 0.001). There were no statistical differences among mean neutralization levels in the 2-, 3-, 4-, and 5-dose groups (*P* > 0.05). Within each immunocompetent dose group, linear mixed models with patient ID as a random effect were used to compare Wuhan, Omicron, SARS-CoV, and W1V1-CoV pseudovirus neutralization levels. For all within-dose group comparisons of vaccinated (2–5 doses) immunocompetent participants (linear mixed model with Tukey HSD test; *P* < 0.001; Supplementary Figure 6b).

## Discussions

As of May 2023, COVID-19-related health emergency restrictions were lifted across the globe [5, 22]. However, SARS-CoV-2 infections continue to be recorded worldwide [6]. This situation has been attributed to the ability of the virus to evade host immune responses including neutralizing antibody-derived immunity. The vast majority of antibody escape mutations have been associated with the S1 subunit of the spike protein especially the Receptor Binding Domain (RBD) but also the N-terminal Domain (NTD) [14–16, 18]. The other region of the spike, the S2 subunit, is the most conserved region amongst coronaviruses [23]. We hypothesized that S2-specific antibody responses are suboptimal in vaccinated and SARS-CoV-2 infected patients resulting in an ineffective neutralization of distant coronaviruses.

Homology between the spike proteins of wild-type Wuhan and the distant SARS coronavirus (SARS-CoV) and the coronavirus of bat origin (W1V1-CoV) have been previously reported at 75.6% and 76.5% [21], respectively. Booster doses induced efficient neutralization of the wild-type Wuhan and the Omicron variants [21]. Our data were in accordance with these findings; booster immunizations provided higher neutralization potency against Wuhan and the Omicron variant while the distant relatives W1V1-CoV and SARS-CoV were less sensitive to serum antibodies obtained from patients who received Wuhan-trimer based immunogens [21]. This weak neutralization of distant coronaviruses was observed despite the S2 region of Wuhan and Omicron sharing an almost 90% identity with SARS-CoV and W1V1-CoV. We correctly predicted that S2-specific antibody levels are suboptimal in vaccinated COVID-19 patients as confirmed by the lower S2-specific antibody levels in comparison with S1-specific antibody levels.

Furthermore, we evaluated the benefit of booster immunization on antibody responses in immunocompromised individuals. Even though we found elevated levels of spike-specific antibody responses in immunocompromised individuals, these levels were lower than that observed among immunocompetent individuals. The presence of significantly lower neutralizing antibody titers for an immunocompromised patient have been previously observed [16]. A contrasting result has also been reported with similar levels of S1-specific antibodies observed between immunocompetent counterparts and a group of 584 immunocompromised patients with hematologic cancers who received a third COVID_19 mRNA vaccine booster [24]. Moreover, the benefit of a third vaccine booster was highlighted in another study which found elevated humoral immune responses in immunocompromised children who had earlier received a second booster vaccine [25].

More recently, the immunocompromised were found to have diminished SARS-CoV-2-specific humoral by comparison with the immunocompetent in a large cohort of 56 immunocompromised participants and 184 non-immunocompromised participants [10]. Our data are in accordance with these later findings. We found lower neutralization antibody potency for immunocompromised individuals versus immunocompetent.

## Limitations

Our study is limited by modest sample sizes, particularly among the immunocompromised sub-group, which may affect our ability to differentiate antibody responses between groups. Another limitation to our study is the absence of samples from vaccinated but non-infected participants. We were unable to discriminate between vaccine induced-immunity and natural immunity following SARS-CoV-2 infection in the current study which only measured spike/spike subunit-specific antibody responses. Future studies should include the measurement of anti-nucleocapsid titers, as this protein is not included in Wuhan spike trimer-based mRNA vaccine considered in this study.

In summary, we found that S1-specific antibody levels were higher in vaccinated and infected COVID-19 patients. We also found that both S1- and S2- specific antibody responses generally correlated with the four coronaviruses (SARS-CoV-2 Wuhan, Omicron, SARS-CoV and W1V1-CoV). Among the immunocompromised, the most distinctive result was obtained with S2-Wuhan-specific antibody levels which correlated with the neutralization capacity against Omicron, SARS-CoV and W1V1-CoV.

### Perspectives:

Our data raises the hope that S2-targeting immunogens can successfully eliminate the spread of SARS-CoV-2 variants [26–28]. The data encourages further efforts towards the development of S2-targeting immunogens as universal vaccines for the eradication of SARS-CoV-2 and the prevention of future excursion of coronaviruses into human populations.

## Methods

### Study enrollment and sample collection.

Serum samples were obtained from the prospective Post-Vaccination Viral Characteristics Study (POSITIVES) between January 2021 and May 2023. These samples were collected between 14 days and 48 days from the first PCR test with a median of 20 days, an interquartile range of 7 days and 75% of samples being under 24 days. Here, we consider the timing of the last dose in our analysis of the impact of the vaccine type, Moderna or Pfizer/BioNTech COVID-19 mRNA. The study has been described in full detail previously [10, 19]. In summary individuals in the Mass General Brigham medical record system with confirmed COVID-19 infection were recruited by phone to join the study. Consenting individuals provided nasal swabs for estimation of virologic decay and blood at enrollment and days 14, 180, and 360. Demographic (sex, age and ethnicity) and vaccination status were obtained from participant reports and medical record abstraction. The immunocompromised individuals in this study have been previously described [10] and they included non-severe immunocompromised participants as well as severe immunocompromised participants [10]. The severe immunocompromised were individual with severe-hematological malignancy/transplant patients (S-HT) and severe autoimmune patients (S-A, participants with autoimmune condition receiving B-cell targeting agents or B cell deficiency) as previously categorized and (NS) [29, 30].

### Ethics declaration.

This study was approved by the Institutional Review Boards of Boston College (IRB Protocol Number # 21.115.01e) and Mass General Brigham (IRB# 2021P000812). Informed consent was obtained from all participants. The authors confirm that all research was performed in accordance with relevant guidelines/regulations.

### Cell Lines.

The 2 cell lines used in this study, Human Kidney Embryonic cells (HEK293T) and HEK293T cells engineered to express the Angiotensin Convertase Enzyme 2 (293T-ACE2) have previously been described [20, 31]. HEK293T-ACE2 cells were a gift from Dr. Huihui Mou and Dr. Michael Farzan (SCRIPPS Research Institute, Florida, USA).

### SARS-CoV-2-specific antibody measurement by ELISA.

#### ELISA Antigens

Soluble SARS-CoV-2 spike S1 and S2 corresponding to the Wuhan and Omicron (B.1.1.529) variants were obtained from Acro Biosystem. Wuhan version of SARS-CoV-2 spike S1 protein, His Tag (Acro Biosystem catalog # S1N-C52H3) contains AA Val 16 - Arg 685 (Accession # QHD43416.1). Wuhan version of SARS-CoV-2 spike S2 protein, His Tag (Acro Biosystem catalog # S2N-C52H5) contains AA Ser 686 – Pro 1213 16 - Arg 685 (Accession # QHD43416.1). Omicron/BA.1 version of SARS-CoV-2 Spike S2, His Tag (Acro Biosystem catalog # S2N-C52Hf) contains AA Ser 686 - Pro 1213 (Accession # QHD43416.1 (N764K, D796Y, N856K, Q954H, N969K, L981F, F817P, A892P, A899P, A942P, K986P, V987P). Mutations are identified on the SARS-CoV-2 Omicron variant (Pango lineage: BA.1; GISAID clade: GRA; Nextstrain clade: 21K). SARS-CoV-2 spike S1, His Tag (B.1.1.529/Omicron) (S1N-C52Ha) contains AA Val 16 - Arg 685 (Accession # QHD43416.1 (A67V, HV69-70del, T95I, G142D, VYY143-145del, N211del, L212I, ins214EPE, G339D, S371L, S373P, S375F, K417N, N440K, G446S, S477N, T478K, E484A, Q493R, G496S, Q498R, N501Y, Y505H, T547K, D614G, H655Y, N679K, P681H)). The spike mutations are identified on the SARS-CoV-2 Omicron variant (Pango lineage: B.1.1.529; GISAID clade: GR/484A; Nextstrain clade: 21K).

#### S2 Antigen modification for solubility

Proline substitutions (F817P, A892P, A899P, A942P, K986P, V987P) were introduced in both spike S2 (Wuhan and Omicron versions) by the manufacturer (Acro Biosystem) in order to prevent the formation of aggregates in the course of protein production.

#### ELISA Procedure

ELISA was performed as previously described [11, 20, 32]. Briefly, 96-well Nunc MaxiSorp ELISA plates (Thermo Scientific) were coated with viral antigens (Wuhan S1, Wuhan S2, Omicron S1 or Omicron S2) diluted in carbonate-bicarbonate buffer to a concentration of 1 mg/mL before incubation for 1 h at room temperature. Plates were washed with a buffer consisting of 50 mM Tris (pH 8.0) (ThermoFisher), 140 mM NaCl (MilliporeSigma), and 0.05% Tween-20 (ThermoFisher). Next, plates were incubated with a blocking buffer consisting of 1% BSA (MilliporeSigma), 50 mM Tris (pH 8.0), and 140 mM NaCl for 30 min at room temperature. The plates were washed 1–3 times after blocking. Serum samples were diluted 1:100 with a dilution buffer consisting of 1% BSA, 50 mM Tris (pH 8.0), 140 mM NaCl, and 0.05% Tween-20. After sample addition, plates were incubated at 37°C for 30 minutes followed by washing, 5 times. Serum IgG levels were detected by addition of HRP-conjugated anti-human-IgG purchased from ThermoFisher (catalog # 62–8420) and diluted (1:4,000). The plates were incubated for 30 min at room temperature. After the washes, TMB substrate (ThermoFisher) was added to each plate for 10 min and the reaction was terminated with TMB stop solution (Southern Biotech). Data were acquired by spectrophotometry at 450 nm using a Victor X5 microplate reader (Perkin Elmer).

### SARS-CoV-2 Pseudovirus Production.

Pseudovirus production and titration have previously been described [11, 20, 32]. The plasmids obtained from Addgene were gifted by Dr. Alejandro Balazs. A group of 4 plasmids - pHAGE-CMV-luc2-IRES-ZsG-W (Addgene plasmid # 164432), pRC-CMV-Rev1b (Addgene plasmid # 164443), pHDM-Tat1b (Addgene plasmid # 164442), pHDM-Hgpm2 (Addgene plasmid # 164441) - were used for production of all pseudovirus variants. Only the plasmid corresponding to the spike differed for the 4 viruses. The plasmids pTwist-SARS-CoV-2 Δ18 (Addgene plasmid # 164436), pTwist-SARS-CoV-2 Δ18 B.1.1.529 (Addgene plasmid # 1789907), pTwist-W1V1-CoV Δ18 (Addgene plasmid # 164439), and pTwist-SARS-CoV Δ18 (Addgene plasmid # 169465) were used for production of the Wuhan, Omicron, SARS-CoV and W1V1-CoV pseudoviruses, respectively. A total of 5 plasmids were therefore used for each of the 4 pseudoviruses with the spike expression plasmid being the only variable. On the day before transfection, 12–15 million HEK293 T cells were seeded in T175 (ThermoFisher) in presence of 25 ml of DMEM10. Before transfection, culture media was replaced with a fresh 25 ml DMEM10. The transfection was performed with GenJet (SignaGen Laboratories) according to the manufacturer’s recommendations. Twenty-four hours later, transfection media was replaced with fresh DMEM10 and culture supernatant containing secreted pseudoviruses was harvested 5 days post-transfection and cleared using a 0.45 μm Nalgene syringe filter (ThermoFisher). The pseudovirus preparation was divided into 1 ml aliquots per cryovial and stored at −80°C.

### SARS-CoV-2 Pseudovirus Titration.

Titration of pseudovirus preparations has been previously described [11, 20, 32]. Here, 293T-ACE2 cells (10^4^ cells/well) were seeded in 100 μl of DMEM10 into 96-well black/clear bottom plates purchased from ThermoFisher (catalog # 165305). For titration, 50 μl of 2x serially diluted pseudovirus preparation were added to corresponding wells. Control (background) wells received 50 μl of DMEM10. On the fifth day, Pseudovirus infectivity was quantified by luciferase assay using the previously described in-house luciferin buffer [11, 33]. Assay plates were read using a Victor X5 microplate reader (Perkin Elmer).

### SARS-CoV-2 Pseudovirus neutralization assay.

Pseudovirus neutralization has previously been described [11, 20, 32]. All reagents, cells, virus and serum were added in a single streamline with incubation and assay readout in the same plate, ThermoFisher 96-well black/clear bottom plates. A luciferase readout of 30,000 luminescence rate units (LRU) was targeted as viral input with a 5-day incubation period. Patients’ sera were diluted with DMEM10 starting at 10-fold dilution and performing 3-time serial dilutions (from 1/10 to 1/21870). A starting dilution of 20x (1/20 to 1/43740) and 30x (1/30 to 1/65610), when necessary, were applied to the samples for which the 10-fold dilutions were insufficient to cross the 50% neutralization mark. Fifty μl of pseudovirus preparations were added onto the diluted sera and the mixtures were incubated for 1 hour at 37°C before addition of HEK293T-ACE2 cells (10^4^ cells/well) prepared in 50 μl of DMEM10. Background wells containing cells-only were prepared while cells plus virus-only (no sera) were prepared as positive controls corresponding to 100% assay readout. The plates were incubated at 37°C, 5% CO2 and 70% humidity for 5 days. Following transduction, cells were lysed and luciferase assay performed as previously described [11, 20, 33]. Fifty microliters of luciferin buffer containing 20 mM Tris-HCl (ThermoFisher), 100 mM EDTA (ThermoFisher), 1 mM MgCl_2_ (ThermoFisher), 26.5 mM MgSO_4_ (ThermoFisher), 17 mM dithiothreitol (Goldbio), 250 mM Adenosine-5’-Triphosphate (Goldbio), 750 mM D-luciferin (Goldbio), were added to the well and incubated for 5 minutes with agitation before luminescence was quantified within 30 minutes of buffer addition using a Victor X5 microplate reader (Perkin Elmer). Neutralization curves were analyzed using GraphPad prism. Neutralizing antibody responses (NT50) were calculated by taking the inverse of the 50% inhibitory concentration value for each sample. Of note, the inverse serial dilution number was multiplied by two to obtain the final NT50 values because (diluted) sera were further diluted with equal volumes of pseudovirus during the serum-virus incubation step.

### Statistical analysis.

Graphpad Prism 9 (v9.3.1) was used to analyze neutralization data and determine the 50% neutralization titer (NT50). R (v4.2.1) was used for all other statistical analyses. Antibody binding means were analyzed using *t*-tests when comparing two groups (e.g., S1- vs S2-specific binding), with a paired test when the same patients were sampled in both groups. Neutralizing antibody titer means were compared using a one-way ANOVA when all samples were independent, and linear mixed models with patient ID as a random effect when the same participant was measured for multiple antigens/pseudoviruses. Due to considerable skew in NT50 values, in these analyses these values were transformed (log10 NT50+1). For significant ANOVA and linear mixed models, differences among groups were identified with the post-hoc Tukey HSD test. Linear correlations between antibody binding and neutralization were analyzed using Pearson’s correlation test. Across all tests an alpha of 0.05 was used to determine statistical significance.

## Figures and Tables

**Table 1.** Demographic and clinical characteristic of patients

	Total	0 Doses	1 Dose	2 Doses	3 Doses	4 Doses	5 Doses
**Patients (n=87)**							
Female (n=60), %	68.976	5.747	0	8.046	34.483	10.345	10.345
Male (n=27), %	31.034	3.448	1.149	2.299	10.345	8.046	5.747
**Last Shot, %**							
Johnson & Johnson (n=1), %	1.149	0	1.149	0	0	0	0
Pfizer (n=40), %	45.977	0	0	6.897	24.138	6.897	8.046
Moderna (n=38), %	43.678	0	0	3.448	20.69	11.494	8.046
None (n=8), %	9.195	NA	NA	NA	NA	NA	NA
**Race %**							
White (n=69), %	79.31	6.897	1.149	6.897	36.782	13.793	13.793
Asian (n=2), %	2.299	0	0	0	2.299	0	0
Black or African American (n=6), %	6.897	0	0	1.149	2.299	2.299	1.149
Others and unknown (n=10), %	11.494	2.299	0	2.299	3.448	2.299	1.149

## Data Availability

Data presented in this study are available by request to the corresponding author.
